# Left Atrial Enlargement in Primary Cryptogenic Strokes Without Atrial Fibrillation

**DOI:** 10.7759/cureus.75084

**Published:** 2024-12-04

**Authors:** Hytham Rashid, Cecilia Pham, Jonathan Brown, Tushar Pansuriya, Negar Niknam, Shai Ring, Aswin Srinivasan, Zuhair Ali, Siva T Sarva, Syed A Raza

**Affiliations:** 1 Cardiovascular Disease, HCA Houston Healthcare, Kingwood, USA; 2 Biomedical Sciences, Tilman J. Fertitta Family College of Medicine at the University of Houston, Houston, USA; 3 Internal Medicine, HCA Houston Healthcare, Kingwood, USA; 4 Graduate Medical Education, HCA Houston Healthcare, Kingwood, USA; 5 Pulmonary and Critical Care Medicine, HCA Houston Healthcare, Kingwood, USA

**Keywords:** atrial cardiopathy, cryptogenic strokes, lae, left atrial enlargement, pcs, strokes

## Abstract

The relationship between left atrial enlargement (LAE) and primary cryptogenic stroke (PCS) remains a mystery. LAE has been proposed to be an independent risk factor of PCS, recurrent ischemic strokes, paroxysmal atrial fibrillation, and thromboembolism. Our study evaluates the prevalence of LAE among patients with PCS in the absence of atrial fibrillation, unlike previous studies that included atrial fibrillation, in order to isolate LAE as a risk factor. We hypothesize there is a direct correlation between the prevalence of LAE and the incidence of PCS. Our multi-center, retrospective, cross-sectional study constructed a database of 646 patients identified with a diagnosis of cerebral infarction over a three-year period. Detailed chart review excluded all patients with known etiologies for stroke, including atrial fibrillation, atrial flutter, prior stroke, systolic heart failure, carotid artery stenosis, patent foramen ovale, thromboembolic disease, previous anticoagulation, or an active cancer diagnosis. Diagnosis of LAE utilized a composite of criteria for transthoracic echocardiogram measurements, including left atrial diameter (LAD) and left atrial volume index (LAVI). All study criteria were met by 154 patients (24%) for analysis, where baseline characteristics included: 79 (51%) male, 104 (67.5%) Caucasian ethnicity, 108 (70%) diagnosed of hypertension (HTN), 80 (52%) previous or current tobacco users, and 47 (31%) diagnosed of diabetes (DM). We utilized logistic regression modeling to examine correlations in our population. Our preliminary analysis found that 74 (48%) patients met at least one criterion for LAE. The mean LAD for patients with and without LAE was 4.1 cm and 3.4 cm, respectively (SD 0.87 vs 0.55, p<0.0001). The mean LAVI for patients with and without LAE was 29.68 mL/m^2^ and 18.44 mL/m^2^, respectively (SD 7.37 vs 5.13, p<0.0001). Our findings support the significance of LAE as a risk factor for cases of PCS. Multiple risk factors were identified in our study population that reflect the importance of preventative counseling for patients with HTN, hyperlipidemia, history of tobacco use, and DM. Routine screening for LAE in patients who suffer a PCS will encourage additional research that may elucidate the clinical relevance of identifying LAE in PCS. For example, whether LAE alone or in the setting of specific comorbidities warrants universal screening practices such as closer monitoring of arrhythmias such as paroxysmal atrial fibrillation to initiate anticoagulation earlier. Additionally, randomized control trials are necessary to determine whether prophylactic anticoagulation reduces future stroke risk among patients identified with LAE.

## Introduction

Stroke is the world's second leading cause of death [1]. When considering both death and disability together, it is the third leading cause globally. Identifying stroke risk factors can optimize prevention strategies and improve outcomes. Ischemic stroke prevention focuses on patients with known risk factors for strokes, including carotid artery stenosis, atrial fibrillation (AF), patent foramen ovale, previous transient ischemic attack or cerebrovascular accident, pulmonary embolism, venous thromboembolism, and hypercoagulable states [2]. However, up to one-third of ischemic strokes occur without identifiable risk factors. These strokes are known as embolic strokes of undetermined source or primary cryptogenic strokes (PCS) [3]. The incidence of cryptogenic strokes varies greatly, with estimates of up to 40% of new strokes [4,5]. This high incidence warrants further research to identify novel risk factors associated with PCS [3].

Left atrial enlargement (LAE) is one such risk factor implicated in PCS. LAE is commonly found in patients with cardiovascular diseases (CVD), such as hypertension (HTN) and valvular heart disease. It is also found in patients with longstanding AF, where it presents a paradox as LAE can also lead to AF. LAE occurs when the atrium remodels in response to stress and increased volume [6]. LAE is defined by imaging in prior studies on echocardiogram, CT and MRI. Both AF and LAE can disrupt its essential role in maintaining optimal blood flow and pressure, leading to blood stasis and thromboembolism formation which can travel from the left atrium to the brain to cause a stroke [7-9].

Previous studies have linked LAE severity to PCS, recurrent ischemic strokes, and paroxysmal AF [7, 10]. While LAE has been linked to the detection of AF in patients with PCS, the relationship between LAE and PCS without AF is not well understood. It is well-recognized that patients with AF may have normal sinus rhythms upon presentation with PCS. This is evident by a prior study showing non-sinus contraction of the left atrial appendage consistent with AF despite being in sinus rhythm [11]. This could suggest that the anatomic remodeling of LAE contributes directly to left atrial thromboembolism, causing PCS, rather than requiring AF. However, identifying AF as a cause of stroke is challenging and often paroxysmal, as demonstrated by a previous study involving patients who exhibited no AF on more than 90% of days following a thromboembolic event [12].

The relationship between LAE and PCS has important clinical implications. Identifying LAE in patients with CVD should prompt further investigation for the presence of AF and other risk factors for stroke, such as HTN and diabetes mellitus (DM). Previous research supports that LAE should be considered an independent risk factor for stroke that may affect future optimal treatment and management strategies for stroke prevention [13]. To the best of our knowledge, no studies were conducted in the United States at the time of our study to assess the relative prevalence and incidence of LAE in stroke patients without AF. Therefore, our study explores the prevalence of LAE in a population of hospitalized patients with a PCS who do not have AF by identifying the incidence of LAE on transthoracic echocardiogram (TTE). Secondary objectives included characterizing the severity of LAE on TTE by evaluating measurements of left atrial diameter (LAD), left atrial volume (LAV), and left atrial volume index (LAVI). Furthermore, our secondary objectives also aimed to identify additional variables influencing PCS risk in LAE through patient-specific demographics utilizing a covariate analysis of potential comorbidities.

## Materials and methods

Study design

In accordance with the STROBE guidelines, we used specific criteria to select patients for inclusion or exclusion for this retrospective cross-sectional study [14]. Our multi-center study evaluated over 5,000 medical records from the HCA Healthcare database, which were collected using specific International Classification of Diseases (ICD) codes and Current Procedural Terminology (CPT) codes to evaluate TTE reports. We also searched the HCA Healthcare database for a list of search terms related to LAE and PCS. The list of ICD and CPT codes are listed in Appendix A and Appendix B. The investigators extracted patient records spanning from January 1st, 2017, to December 31st, 2020.

Upon obtaining ECG readings from the HCA medical users software exchange (MUSE) application for inpatient status ECGs, the following search terms were used to identify LAE: “left atrial volume” OR “left atrial size” OR “left atrial dimension” OR “left atrial diameter” OR “left atrial dilatation” OR “left atrial hypertrophy” OR “left atrial enlargement” OR “left atria volume” OR “left atria size” OR “left atria dimension” OR “left atria diameter” OR “left atria dilatation” OR “left atria hypertrophy” OR “left atria enlargement” OR “left atrium volume” OR “left atrium size” OR “left atrium dimension” OR “left atrium diameter” OR “left atrium dilatation” OR “left atrium hypertrophy” OR “left atrium enlargement” OR “enlarged left atrium” OR “enlarged left atria” OR “heart atrium enlargement” OR “atriomega” AND “cerebral embolism” OR stroke OR “Apoplexy” OR “Brain Vascular Accident” OR “CVA” OR “Cerebrovascular Accident*” OR “ischemic infarction” OR “embolism” OR “thromboembolism” OR “thrombosis”.

**Figure 1:**
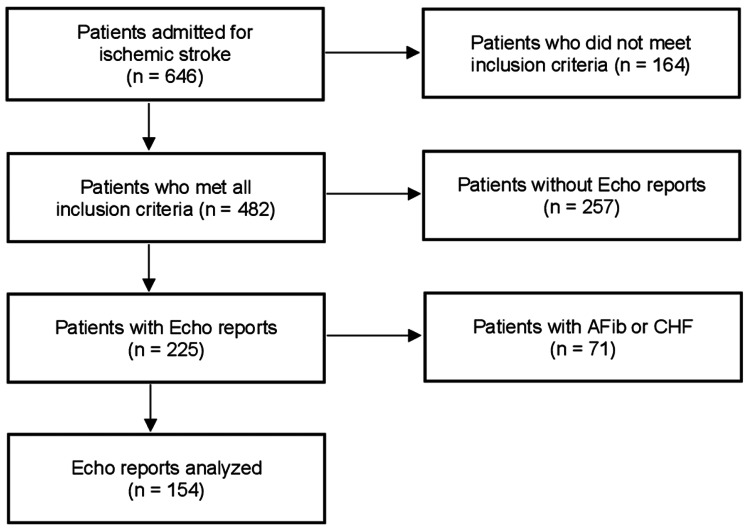
Inclusion and exclusion per STROBE guidelines.

All reports were hand-reviewed by two independent analysts to de-identify data prior to analysis. Patients with records of magnetic resonance imaging (MRI) reports confirming acute cerebral infarction were included. Patients who were diagnosed with LAE were also included, and a composite of criteria for TTE measurements, including LAD and LAVI, were used. Patients without an MRI for stroke confirmation were excluded. Patients with known etiologies for PCS were also excluded. Examples include AF, atrial flutter, prior stroke, PFO, systolic heart failure, carotid artery stenosis, thromboembolic disease, previous anticoagulation, or an active cancer diagnosis. The identification, inclusion, and exclusion processes are summarized in Figure 1.

The normal range values for LAD, LAV, and LAVI that were used as the standard values are provided in Table 1. These standard values are based on the 2015 recommendations for cardiac chamber quantification by echocardiography in adults from the American Society of Echocardiography (ASE) and the European Association of Cardiovascular Imaging [15]. These values were utilized in order to assess discrepancies between the various metrics for identifying LAE, which was then utilized to construct discrete tables identifying shifts in the severity of LAE depending on the metrics. Modifiable risk factors in this study included smoking status, along with medical histories significant for HTN, DM, hyperlipidemia (HLD), and obesity. Risk factors were stratified by severity using ICD codes (Appendix A and Appendix B). Nonmodifiable risk factors included genetic predispositions to hypercoagulable disorders, race, gender, and age.

**Table 1:** Standardized ranges for echocardiographic parameters. LAD: left atrial diameter; LAV: left atrial volume; LAVI: left atrial volume index.

Parameters	Gender	Normal	Mild	Moderate	Severe
LAD (cm)	Male	≤4.0	4.1-4.6	4.7-5.1	≥5.2
	Female	≤3.8	3.9-4.2	4.3-4.6	≥4.7
LAV (mL)	Male	18-58	59-68	69-78	≥79
	Female	22-52	53-62	63-72	≥73
LAVI (mL/m²) (2005)	Both	16-28	29-33	34-39	≥40
LAVI (mL/m²) (2015)	Both	16-34	35-41	42-48	≥48

Power calculation

Drawing from prior literature, a 10% relative incidence of LAE was documented within the general populations of Poland, Germany, and Japan [16-18]. Given this, the researchers anticipated there would be a higher incidence of LAE in a hospitalized population with acute strokes. Therefore, the expected LAE incidence was set to be 20%. We calculated the minimum necessary sample size to be 85 patients to achieve a statistical power of 80%. As the thresholds for LAE measurements have increased, an 80% power was chosen to avoid including borderline cases of LAE that are no longer representative of the newer guidelines for diagnosis.

Statistical analysis

This retrospective cross-sectional study identified two separate cohorts based on the presence or absence of LAE. We compared them across a variety of demographics as well as baseline echocardiographic characteristics to determine if there was any correlation, such as increased incidence or representation of certain demographics. Based on these results, we identified the severity of LAE in patients who met certain criteria or metrics. Patient characteristics that were evaluated include age, smoking status, payer status, race, ethnicity, biological sex, body mass index (BMI), DM and HTN. The statistical analysis approach utilized to examine the association between LAE and patient characteristics was logistic regression modeling. Based on preliminary analysis, our statisticians calculated odds ratios with 95% confidence intervals for the variables of interest due to the lack of a true control group in our retrospective study. Reciprocal odds ratios were calculated by dividing one by the original odds ratios and confidence intervals. An alpha level of 0.05 was used to determine significant associations. Data analysis was conducted using SAS version 9.4 (SAS Institute Inc., Cary, North Carolina, USA). SAS was selected because it is the available software for the hospital system.

Bias

Retrospective studies involve identifying patients who meet the defined inclusion criteria. An inherent selection bias is present in our study due to the selection of patients who have experienced strokes. Typically, such patients have underlying risk factors that increase the likelihood of LAE. While the methodology of the study did not directly address the selection bias, its significance is discussed within the limitations outlined in the Discussion section. Our study accounted for sampling bias by including only patients admitted for PCS while excluding those with known risk factors. This approach helps prevent the selection of patients with alternative stroke causes, thereby reducing confounding factors. It also avoids the bias of selecting either sicker or healthier patients.

## Results

Our study retrospectively identified 646 patients admitted with a diagnosis of PCS to be assessed for eligibility, resulting in 482 patients (75%) who met all inclusion criteria. However, echocardiography reports were available for only 225 (47%) of these patients. Among these patients, 54% were female, 67% were of Caucasian ethnicity, 60% had a diagnosis of HTN, 45% were previous or current tobacco users, and 31% had a diagnosis of DM. Patients with a history of atrial fibrillation (AF) or congestive heart failure were excluded, forming the final study population of 154 patients (24%) who fulfilled all study criteria for analysis, shown in Table 2. Baseline characteristics included 51% males, 67.5% with Caucasian ethnicity, 70% with a diagnosis of HTN, 52% with a history of or current tobacco use, and 31% with a diagnosis of DM. The clinical significance of these findings correlates with the known risk factors we expected to be represented in our population of stroke patients.

**Table 2:** Baseline characteristics of the patient population. HLD: hyperlipidemia, DM: diabetes mellitus, HTN: hypertension, HF: heart failure.

Variables		Statistical parameter	Initial population (n=646)	Final population (n=154)
						Age	N (%)	646 (100%)	154 (100%)
	(Years)	Mean±SD	66.09±5.20	64.88±13.82
	NIH Stroke Score	N (%)	376 (58.2%)	96 (62.3%)
		Mean±SD	8.71±8.28	8.03±8.80
Ethnicity	Hispanic	N (%)	97 (15.0%)	27 (17.5%)
	Non hispanic	N (%)	511 (79.1%)	115 (74.7%)
	Unknown	N (%)	38 (5.9%)	12 (7.8%)
Race	Black	N (%)	93 (14.4%)	21 (13.6)
	Other	N (%)	86 (13.3%)	19 (12.3%)
	Unknown	N (%)	31 (4.8%)	10 (6.5%)
	White	N (%)	436 (67.5%)	104 (67.5%)
Sex	Female	N (%)	350 (54.2%)	75 (48.7%)
	Male	N (%)	296 (45.8%)	79 (51.3%)
DM		N (%)	199 (30.8%)	47 (30.5%)
HTN		N (%)	390 (60.4%)	108 (70.0%)
HLD		N (%)	373 (57.7%)	8 (5.2%)
Smoker		N (%)	291 (45.1%)	80 (52.0%)
Diastolic HF		N (%)	32 (5.0%)	4 (2.6%)
Expired/hospice		N (%)	62 (9.6%)	12 (7.8%)

Additionally, Table 3 presents the baseline echocardiographic findings of the patient population. With the identification of 154 patients for our study, we were able to achieve a study power of approximately 95%. This higher level of power implies that our population had a larger prevalence of LAE than expected. This may correlate with our expectation that there would be a higher incidence of LAE in hospitalized patients than in the general population. 

**Table 3:** Baseline echocardiographic findings of the patient population. LAD: left atrial diameter; LAV: left atrial volume; LAVI: left atrial volume index, EF: ejection fraction.

EF (%)	N	129
	Mean±SD	61%±9.43%
LAD	N	140
	Mean±SD	3.71±0.80
LAV	N	69
	Mean±SD	51.77±16.88
LAVI	N	69
	Mean±SD	26.42±8.43

Statistical analysis was conducted using the Chi-square test and two-tailed T-test to determine the following results. For statistical testing, Chi-square was utilized to compare discrete, categorical values such as race and ethnicity, while T-testing was utilized to compare continuous, numeric values such as age and NIH Stroke Score (NIHSS). As our study achieved a 95% power, it is possible that our study population had a higher prevalence than expected of stroke and, in turn, potentially LAE; however, further research would be needed to assess this in a larger population.

Table 4 displays the distribution of echocardiographic findings based on the presence of LAE. The mean LAD for patients with and without LAE was 4.1 cm and 3.4 cm, respectively (SD 0.87 vs 0.55, p<0.0001). Similarly, the mean LAVI for patients with and without LAE was 29.68 mL/m^2^ and 18.44 mL/m^2^, respectively (SD 7.37 vs 5.13, p<0.0001).

**Table 4:** Distribution of echocardiographic findings based on the presence of LAE. LAE: left atrial enlargement, LAD: left atrial diameter; LAV: left atrial volume; LAVI: left atrial volume index; HF: heart failure.

Variables	Statistical parameter	No LAE (n=80)	LAE (n=74)	Test	p-value
LAD (cm)	N (%)	72 (90.0%)	68 (91.9%)	T-Test	<0.0001
	Mean±SD	3.35±0.55	4.08±0.87		
LADI (cm/m²)	N (%)	71 (88.8%)	67 (90.5%)	T-Test	<0.0001
	Mean±SD	1.73±0.29	2.15±0.54		
LAV (mL)	N (%)	20 (25.0%)	49 (66.2%)	T-Test	<0.0001
	Mean±SD	36.51±9.74	58.00±15.34		
LAVI (mL/m²)	N (%)	20 (25.05%)	49 (66.2%)	T-Test	<0.0001
	Mean±SD	18.44±5.13	29.68±7.37		
Diastolic HF	N (%)	2 (2.50%)	2 (2.70%)	Chi-Square	0.937

The significance of demographics and comorbidities was assessed through calculations detailed in Tables 5 and Table 6. The tables examine whether various comorbidities and demographic considerations correlate with any statistically significant differences between patients with and without LAE. There was a statistically significant difference in regards to age, which likely comments on the natural development of LAE with cardiac remodeling as patients age, as seen in the average age of 62.86±13.54 years in patients without LAE versus 67.05±13.99 years in patients with LAE (p*=*0.0305). There was also a statistically significant difference in regards to NIHSS, as seen in the average NIHSS of 9.56±10.19 in patients without LAE versus 6.37±6.84 in patients with LAE (p=0.0365). This indicates that patients presenting with strokes due to LAE tend to present with milder strokes despite advanced age. Furthermore, it is possible that the nature of the emboli leading to strokes may differ in size in patients with LAE, and this may, in turn, determine the relative severity of the stroke. There were no other clear correlations between other medical conditions, such as DM, HTN, or HLD, nor in terms of payer status, ethnicity, race, or sex when considering the presence or absence of LAE. This is seen in the p-values, which were calculated based on the test chosen for the type of data being evaluated. This may indicate that LAE is prevalent independently of comorbidities and socio-economic considerations. However, further research would need to be conducted to elucidate this possibility.

**Table 5:** Distribution of comorbidities based on the presence of LAE. HTN: hypertension; DM: diabetes mellitus; HLD: hyperlipidemia; EF: ejection fraction.

Comorbidities	Statistical parameter	No LAE (n=80)	LAE (n=74)	Test	p-value
						Diastolic HF	N (%)	2 (2.5%)	2 (2.7%)	Chi-Square	0.937
DM	N (%)	26 (32.5%)	21 (28.4%)	Chi-Square	0.5789
HTN	N (%)	55 (68.8%)	53 (71.6%)	Chi-Square	0.6973
HLD	N (%)	4 (5.0%)	4 (5.4%)	Chi-Square	0.9098
Smoker	N (%)	39 (48.8%)	41 (55.4%)	Chi-Square	0.4089
Expired/Hospice	N (%)	7 (8.8%)	5 (6.8%)	Chi-Square	0.6448
EF (%)	N (%)	62 (77.5%)	67 (90.5%)	T-Test	0.1477
	Mean±SD	62%±10.3%	60%±8.6%		

The significance of demographics was assessed through calculations detailed in Table 6.

**Table 6:** Distribution of demographics based on the presence of LAE. LAE: left atrial enlargement.

Variables		Statistical parameter	No LAE (n=80)	LAE (n=74)	Test	p-value
								Age	N (%)	80 (100%)	74 (100%)	T-Test	0.0305
	(Years)	Mean±SD	62.86±13.54	67.05±13.99		
	NIH Stroke Score	N (%)	50 (62.5%)	46 (62.2%)	T-Test	0.0365
		Mean±SD	9.56±10.19	6.37±6.84		
Payer Status	Government	N (%)	30 (37.5%)	22 (29.7%)	Chi Square	0.593
	Other	N (%)	42 (52.5%)	44 (59.5%)		
	Uninsured	N (%)	8 (10.0%)	8 (10.8%)		
Ethnicity	Hispanic	N (%)	17 (21.3%)	10 (13.5%)	Chi Square	0.2084
	Not Hispanic	N (%)	55 (68.8%)	60 (81.1%)		
	Unknown	N (%)	8 (10.0%)	4 (5.4%)		
Race	Black	N (%)	13 (16.3%)	8 (10.8%)	Chi Square	0.3863
	Other	N (%)	8 (10.0%)	11 (14.9%)		
	Unknown	N (%)	7 (8.8%)	3 (4.1%)		
	White	N (%)	52 (65.0%)	52 (70.3%)		
Sex	Female	N (%)	43 (53.8%)	32 (43.2%)	Chi Square	0.1924
	Male	N (%)	37 (46.3%)	42 (56.8%)		

Tables 7-10 display the distribution of LAE severity based on parameters such as LAD, LAV, and LAVI. These measurements were obtained following standard protocols for transthoracic echocardiography as set by the ASE. Table 9 and Table 10 present the distribution of LAE severity according to the 2005 and 2015 ASE guidelines on LAVI measurement, respectively. Table 9, which describes the severity of LAE based on the 2015 LAVI guidelines, is the most sensitive parameter for ruling out LAE. However, upon comparing the 2005 and 2015 LAVI guidelines, it was observed that 12 patients were classified as having severe LAE according to the 2005 guidelines, while none met the more stringent 2015 guidelines. 

**Table 7:** Distribution of LAE severity based on LAD. LAD: left atrial diameter; LAE: left atrial enlargement.

Variables		Statistical parameter	Not recorded	Normal	Mild	Moderate	Severe	Total
	Age	N (%)	16 (10.4%)	93 (60.4%)	25 (16.2%)	8 (5.2%)	12 (7.8%)	154 (100%)
	(Years)	Mean±SD	65.75±14.96	64.28±13.85	64.36±11.86	61.38±17.09	71.75±14.42	64.88±13.87
Smoking	No	N (%)	9 (56.3%)	42 (45.2%)	14 (56.0%)	6 (75.0%)	3 (25.0%)	74 (48.1%)
	Yes	N (%)	7 (43.8%)	51 (54.8%)	11 (44.0%)	2 (25.0%)	9 (75.0%)	80 (52.0%)
Diastolic HF	No	N (%)	16 (100%)	91 (97.9%)	24 (96.0%)	7 (87.5%)	12 (100%)	150 (97.4%)
	Yes	N (%)	0 (0%)	2 (2.2%)	1 (4.0%)	1 (12.5%)	0 (0.0%)	4 (2.6%)
Payer Status	Government	N (%)	7 (43.8%)	32 (34.4%)	7 (28.0%)	3 (37.5%)	3 (25.0%)	52 (33.8%)
	Other	N (%)	8 (50.0%)	52 (55.9%)	12 (48.0%)	5 (62.5%)	9 (75.0%)	86 (55.8%)
	Uninsured	N (%)	1 (6.3%)	9 (9.7%)	6 (24.0%)	0 (0.0%)	0 (0.0%)	16 (10.4%)
Ethnicity	Hispanic	N (%)	0 (0.0%)	17 (18.3%)	5 (20.0%)	1 (12.5%)	4 (33.3%)	27 (17.5%)
	Not Hispanic	N (%)	15 (93.8%)	66 (71.0%)	20 (80.0%)	6 (75.0%)	8 (66.7%)	115 (74.7%)
	Unknown	N (%)	1 (6.3%)	10 (10.8%)	0 (0.0%)	1 (12.5%)	0 (0.0%)	12 (7.8%)
Race	Black	N (%)	5 (31.3%)	11 (11.8%)	3 (12.0%)	0 (0.0%)	2 (16.7%)	21 (13.6%)
	Other	N (%)	0 (0.0%)	11 (11.8%)	7 (28.0%)	0 (0.0%)	1 (8.3%)	19 (12.3%)
	Unknown	N (%)	1 (6.3%)	7 (7.5%)	0 (0.0%)	1 (12.5%)	1 (8.3%)	10 (6.5%)
	White	N (%)	10 (62.5%)	64 (68.8%)	15 (60.0%)	7 (87.5%)	8 (66.7%)	104 (67.5%)
Sex	Female	N (%)	4 (25.0%)	49 (52.7%)	9 (36.0%)	4 (50.0%)	9 (75.0%)	75 (48.7%)
	Male	N (%)	12 (75.0%)	44 (47.3%)	16 (64.0%)	4 (50.0%)	3 (25.0%)	79 (51.3%)

The clinical significance of our findings displayed in Tables 8-10 lies in illustrating how shifting the threshold for LAE from the 2005 ASE criteria to the 2015 ASE criteria excluded many of our patients who still developed strokes. These echocardiographic measurements were obtained following standardized protocols as defined by the ASE and explained in detail by the ASE guidelines, as mentioned above. Identifying this shift in prevalence may mean that there are still other underlying risk factors for stroke in our patient population to be researched or that even a small degree of LAE can have a significant correlation with stroke risk. The overall trend observed in our results shows how LAE is present in patients with strokes, independent of all other modifiable and nonmodifiable risk factors, including smoking status, ethnicity, race, and biological sex.

**Table 8:** Distribution of LAE severity based on LAV. LAV: left atrial volume, LAE: left atrial enlargement

Variables		Statistical parameter	Not recorded	Normal	Mild	Moderate	Severe	Total
	Age	N (%)	87 (56.5%)	43 (27.9%)	9 (5.8%)	10 (6.5%)	5 (3.2%)	154 (100%)
	(Years)	Mean±SD	64.57±14.03	65.74±15.06	62.00±7.48	64.50±9.61	68.60±19.41	64.88±13.87
Smoking	No	N (%)	45 (51.7%)	19 (44.2%)	4 (44.4%)	4 (40.0%)	2 (40.0%)	74 (48.1%)
	Yes	N (%)	42 (48.3%)	24 (55.8%)	5 (55.6%)	6 (60.0%)	3 (60.0%)	80 (52.0%)
Diastolic HF	No	N (%)	85 (97.7%)	42 (97.7%)	9 (100%)	10 (100%)	4 (80.0%)	150 (97.4%)
	Yes	N (%)	2 (2.3%)	1 (2.3%)	0 (0.0%)	0 (0.0%)	1 (20.0%)	4 (2.6%)
Payer status	Government	N (%)	31 (35.6%)	14 (32.6%)	3 (33.3%)	1 (10.0%)	3 (60.0%)	52 (33.8%)
	Other	N (%)	48 (55.2%)	26 (60.5%)	4 (44.4%)	7 (70.0%)	1 (20.0%)	86 (55.8%)
	Uninsured	N (%)	8 (9.2%)	3 (7.0%)	2 (22.2%)	2 (20.0%)	1 (20.0%)	16 (10.4%)
Ethnicity	Hispanic	N (%)	21 (24.1%)	2 (4.7%)	2 (22.2%)	2 (20.0%)	0 (0.0%)	27 (17.5%)
	Non-Hispanic	N (%)	60 (69.0%)	37 (86.0%)	5 (55.6%)	8 (80.0%)	5 (100%)	115 (74.7%)
	Unknown	N (%)	6 (6.9%)	4 (9.3%)	2 (22.2%)	0 (0.0%)	0 (0.0%)	12 (7.8%)
Race	Black	N (%)	11 (12.6%)	9 (20.9%)	1 (11.1%)	0 (0.0%)	0 (0.0%)	21 (13.6%)
	Other	N (%)	13 (14.9%)	2 (4.7%)	1 (11.1%)	2 (20.0%)	1 (20.0%)	19 (12.3%)
	Unknown	N (%)	4 (4.6%)	5 (11.6%)	1 (11.1%)	0 (0.0%)	0 (0.0%)	10 (6.5%)
	White	N (%)	59 (67.8%)	27 (62.8%)	6 (66.7%)	8 (80.0%)	4 (80.0%)	104 (67.5%)
Sex	Female	N (%)	44 (50.6%)	21 (48.8%)	4 (44.4%)	4 (40.0%)	2 (40.0%)	75 (48.7%)
	Male	N (%)	43 (49.4%)	22 (51.2%)	5 (55.6%)	6 (60.0%)	3 (60.0%)	79 (51.3%)

**Table 9:** Distribution of LAE severity based on LAVI (2005). LAE: left atrial enlargement.

Variables		Statistical parameter	Not recorded	Normal	Mild	Moderate	Severe	Total
	Age	N (%)	95 (61.7%)	39 (25.3%)	7 (4.5%)	7 (4.5%)	6 (3.9%)	154 (100%)
	(Years)	Mean±SD	64.03±14.40	66.82±12.37	62.57±15.80	61.86±10.35	71.83±16.67	64.88±13.87
Smoking	No	N (%)	52 (54.7%)	14 (35.9%)	2 (28.6%)	3 (42.9%)	3 (50.0%)	74 (48.1%)
	Yes	N (%)	43 (45.3%)	25 (64.1%)	5 (71.4%)	4 (57.1%)	3 (50.0%)	80 (52.0%)
Diastolic HF	No	N (%)	93 (97.9%)	38 (97.4%)	7 (100%)	7 (100%)	5 (83.3%)	150 (97.4%)
	Yes	N (%)	2 (2.1%)	1 (2.6%)	0 (0.0%)	0 (0.0%)	1 (16.7%)	4 (2.6%)
Payer status	Government	N (%)	33 (34.7%)	13 (33.3%)	1 (14.3%)	2 (28.6%)	3 (50.0%)	52 (33.8%)
	Other	N (%)	52 (54.7%)	23 (59.0%)	5 (71.4%)	4 (57.1%)	2 (33.3%)	86 (55.8%)
	Uninsured	N (%)	10 (10.5%)	3 (7.7%)	1 (14.3%)	1 (14.3%)	1 (16.7%)	16 (10.4%)
Ethnicity	Hispanic	N (%)	21 (22.1%)	3 (7.7%)	1 (14.3%)	2 (28.6%)	0 (0.0%)	27 (17.5%)
	Not Hispanic	N (%)	67 (70.5%)	31 (79.5%)	6 (85.7%)	5 (71.4%)	6 (100%)	115 (74.7%)
	Unknown	N (%)	7 (7.4%)	5 (12.8%)	0 (0.0%)	0 (0.0%)	0 (0.0%)	12 (7.8%)
Race	Black	N (%)	12 (12.6%)	9 (23.1%)	0 (0.0%)	0 (0.0%)	0 (0.0%)	21 (13.6%)
	Other	N (%)	14 (14.7%)	2 (5.1%)	0 (0.0%)	2 (28.6%)	1 (16.7%)	19 (12.3%)
	Unknown	N (%)	6 (6.3%)	3 (7.7%)	0 (0.0%)	1 (14.3%)	0 (0.0%)	10 (6.5%)
	White	N (%)	63 (66.3%)	25 (64.1%)	7 (100%)	4 (57.1%)	5 (83.3%)	104 (67.5%)
Sex	Female	N (%)	48 (50.5%)	16 (41.0%)	5 (71.4%)	2 (28.6%)	4 (66.7%)	75 (48.7%)
	Male	N (%)	47 (49.5%)	23 (59.0%)	2 (28.6%)	5 (71.4%)	2 (33.3%)	79 (51.3%)

**Table 10:** Distribution of LAE severity based on LAVI (2015). LAVI: left atrial volume index.

Variables		Statistical parameter	Not recorded	Normal	Mild	Moderate	Severe	Total
	Age	N (%)	94 (61.0%)	50 (32.5%)	4 (2.6%)	6 (3.9%)	0 (0%)	154 (100%)
	(Years)	Mean±SD	64.22±14.45	65.40±12.72	63.25±10.59	71.83±16.67	-	64.88±13.87
Smoking	No	N (%)	52 (55.3%)	18 (36.0%)	1 (25.0%)	3 (50.0%)	-	74 (48.1%)
	Yes	N (%)	42 (44.7%)	32 (64.0%)	3 (75.0%)	3 (50.0%)	-	80 (52.0%)
Diastolic HF	No	N (%)	92 (97.9%)	49 (98.0%)	4 (100%)	5 (83.3%)	-	150 (97.4%)
	Yes	N (%)	2 (2.1%)	1 (2.0%)	0 (0.0%)	1 (16.7%)	-	4 (2.6%)
Payer status	Government	N (%)	33 (35.1%)	14 (28.0%)	2 (50.0%)	3 (50.0%)	-	52 (33.8%)
	Other	N (%)	52 (55.3%)	31 (62.0%)	1 (25.0%)	2 (33.3%)	-	86 (55.8%)
	Uninsured	N (%)	9 (9.6%)	5 (10.0%)	1 (25.0%)	1 (16.7%)	-	16 (10.4%)
Ethnicity	Hispanic	N (%)	22 (23.4%)	4 (8.0%)	1 (25.0%)	0 (0.0%)	-	27 (17.5%)
	Non-Hispanic	N (%)	66 (70.2%)	40 (80.0%)	3 (75.0%)	6 (100%)	-	115 (74.7%)
	Unknown	N (%)	6 (6.4%)	6 (12.0%)	0 (0.0%)	0 (0.0%)	-	12 (7.8%)
Race	Black	N (%)	12 (12.8%)	9 (18.0%)	0 (0.0%)	0 (0.0%)	-	21 (13.6%)
	Other	N (%)	14 (14.9%)	3 (6.0%)	1 (25.0%)	1 (16.7%)	-	19 (12.3%)
	Unknown	N (%)	6 (6.4%)	3 (6.0%)	1 (25.0%)	0 (0.0%)	-	10 (6.5%)
	White	N (%)	62 (66.0%)	35 (70.0%)	2 (50.0%)	5 (83.3%)	-	104 (67.5%)
Sex	Female	N (%)	48 (51.1%)	22 (44.0%)	1 (25.0%)	4 (66.7%)	-	75 (48.7%)
	Male	N (%)	46 (48.9%)	28 (56.0%)	3 (75.0%)	2 (33.3%)	-	79 (51.3%)

The results of the multivariable logistic regression analysis are shown in Table 11. Age and ethnicity were found to be significantly associated with the odds of experiencing LAE. Regarding age, LAE likely correlates to age-related changes that are expected and previously described. Controlling for smoking status, payer status, ethnicity and race, sex, BMI, DM, and HTN, the odds of LAE slightly decreased for every one-year increase in age (OR: 0.966, 95% CI: 0.939-0.994). This finding contrasts previous literature, which states that LAV may increase due to normal aging [19-21] and as a consequence of cardiovascular disease [22, 23]. LAE is also associated with impaired performance of activities of daily living in older individuals [24]. Additionally, our study found that Hispanic patients were 3.861 times as likely to have LAE compared to non-Hispanic patients when controlling for the other variables, including HTN, DM, smoking history, race, ethnicity, sex, and payer status (95% CI: 1.241-12.048). Of note, while Hispanic patients may have higher odds, our study size is too small to generalize, specifically we only had 17 (21.3%) Hispanic-identified patients without LAE vs 10 (13.5%) with LAE. These novel findings warrant further analysis.

**Table 11:** Odds ratio estimates; multivariable logistic regression model predicting LAE. HTN: hypertension; DM: diabetes mellitus; BMI: body mass index.

Variables		Odds ratio	95% Wald confidence intervals	
Payer status	Government vs Uninsured	2.156	0.523	8.885
	Other vs Uninsured	1.133	0.319	4.021
Race	Black vs White	1.965	0.701	5.505
	Other vs White	0.306	0.084	1.116
	Unknown vs White	0.805	0.143	4.544
Ethnicity	Non-Hispanic vs Hispanic	0.259	0.083	0.806
	Unknown vs Hispanic	0.572	0.098	3.324
Sex	Female vs Male	1.648	0.798	3.405
DM	No vs Yes	0.598	0.274	1.303
HTN	No vs Yes	1.012	0.468	2.185
Smoker	No vs Yes	1.575	0.766	3.24
Age		0.966	0.939	0.994
BMI		0.989	0.961	1.017

## Discussion

Our study investigated the correlation between LAE and PCS to ascertain its implications for clinical management. After excluding other risk factors, our findings support LAE as an independent risk factor for PCS in the absence of AF. However, paroxysmal AF could not be excluded due to imperfect measures, and a subsequent diagnosis of AF following the stroke could not be determined. The high prevalence of LAE in our study was consistent with prior studies, such as the atherosclerosis risk in communities (ARIC) study, which followed over 15,000 participants for several years to examine the risk factors for cardiovascular diseases. The ARIC study found that LAE was associated with an increased risk of stroke, even after adjusting for other risk factors [13]. Our findings support the ARIC study in proposing that left atrial size may serve as a more ideal and stable predictor than AF alone in ischemic stroke patients, especially for the multitude of patients without AF diagnosed, as seen in many cryptogenic ischemic strokes. This may be especially true given the cost and low yield of techniques to detect AF that have been described in previous literature [25]. Paroxysmal AF might influence the relationship between LAE and PCS in that it can directly lead to LAE, however as this study was conducted on data from the hospital, access to outpatient rhythm monitoring devices was not available for analysis.

The potential mechanisms through which LAE may contribute to stroke risk are theorized as potentially being due to more turbid flow leading to coagulation within a larger atrium. It is also possible that there is a subsequent reduction in flow out of the left atrial appendage, leading to thrombus formation, which independently increases stroke risk as well. For this reason, individual risk factors, such as the presence of well-known alternative causes of stroke, should be balanced against the use of LAE as an independent predictive marker for stroke. That said, clinicians can integrate the presence of LAE into their decision-making processes, for example, by noting that even though a potential patient with severe LAE may have an otherwise low risk for stroke. For example, when treating a patient with paroxymal AF whose calculated stroke risk is low but who is found to have severe LAE, it may be beneficial to discuss pharmacologic therapy such as prophylactic anticoagulation or antiplatelet therapies. This is especially true as new research is underway to identify the utility of such strategies. It is also important to realize that multiple risk factors were identified in our study population that reflect the importance of preventative counseling for patients with HTN, HLD, a history of tobacco use, and DM. More than two-thirds of the patient population with LAE had HTN, indicating that LAE may serve as a surrogate predictor for the influence of HTN on cardiovascular diseases, including stroke.

While our study supports that LAE may be an independent risk factor for stroke, it is important to note that not all patients with LAE may have a stroke. Treatment and prevention strategies should be individualized based on each patient's specific risk factors and medical history [2]. The stroke prevention in atrial fibrillation or “SPAF” study was a multicenter, randomized, placebo-controlled trial that evaluated the use of anticoagulation with warfarin therapy compared to aspirin to prevent stroke in patients with AF [26]. The SPAF trial found that anticoagulation therapy significantly reduced the risk of stroke in these patients by preventing the formation of blood clots in the left atrium. Several other interventions have been shown to reduce the risk of stroke in patients with LAE by reducing the risk of AF, including rhythm control and blood pressure management [27-29]. Rhythm control strategies, such as cardioversion or catheter ablation, can reduce the risk of AF and subsequent stroke [27]. Lastly, aggressive blood pressure control has been shown to reduce the risk of stroke in patients with LAE and HTN [28].

Study limitations

Generalizability

While our study included 83 facilities over three years, only 646 patients diagnosed with PCS were pulled into our retrospective cohort. This is due in part to the high threshold set by multiple exclusion criteria that are commonly documented in patients with PCS. For example, not all patients had an echocardiogram report available for inclusion, limiting our sample size. Acknowledging this, it is important to note that our study appears overpowered for the high prevalence, which may have provided a positive skew in the overall quality of our data. This means that it is possible our data had more cases of LAE than in the general population.

Documentation

As there is no ICD coding for LAE, retrospective studies relying on coding for inclusion and exclusion practices are inherently limited. Without a code to quickly pull data, LAE is, in effect, an invisible pandemic in cardiovascular studies reporting retrospectively like our study. As Table 5 showed the variation of severity following different metrics of LAE, the current 2015 ASE guidelines for LAE sizing thresholds may need to be adjusted and lowered to identify more cases of LAE. Therefore, validation at other facilities may show variation in reporting practices, as it is possible that documentation and diagnostic coding can vary between institutions, thus affecting study results. Additionally, many facilities did not include LAVI and other echocardiographic measurements, which significantly reduced the analysis of our paper, specifically in regard to tables 7-10. Furthermore, there is no universal terminology for LAE, as some authors report on “atrial cardiopathy” or “atrial dilation” instead. Our study chose to utilize “LAE” based on systematic reviews of similar literature and in accordance with echocardiographic reporting standards. The absence of standardized reporting of ICD codes impacts data collection by reducing the number of patients that were identified and thus analyzed. As this is a common issue in healthcare, the downstream impact on clinical practice is a delay in identifying patterns in healthcare, such as a correlation between LAE and PCS.

Correlation of Echocardiographic Findings

We found that LAE may be an independent risk factor for PCS in patients without a prior history of AF. Therefore, identifying LAE on echocardiograms may be an indication for preventative anticoagulation. However, an echocardiogram cannot assess the complex physiologic changes in cardiac remodeling over time, which may warrant a separate analysis. Furthermore, variation in reporting practices limits the generalizability of our study, but also all studies on the subject until a universal coding system is enforced and practiced,

Retrospective Design

Prospective studies may determine the annual incidence of PCS in patients with LAE. Such a study would if planned far enough ahead include early imaging to measure for the presence of LAE at younger ages, then follow patients for a few decades to observe for the presence of PCS and potential LAE to draw a more robust correlation. This could be done while also tracking metrics such as the length of stay for the hospitalization, mortality, and ICU admissions to provide more robust information on the significance of LAE.

Statistical Analysis

The documentation and data retrieval limitations may have impacted our study’s results, so addressing these issues by utilizing clinician-driven data collection could improve study quality. In contrast, our access was limited to de-identified data that required a third-party to retrieve. This entity was tasked with the removal of all identifying information from reports and imaging and was not a clinician. They were limited by both a lack of medical knowledge and an inability to automate the process due to variations in reporting structure at different facilities and by different providers. The de-identified reports then went to be analyzed by a third-party statistician who was also not a clinician and was unable to obtain all requested data. A glossary of alternate terminology was prepared to be able to identify the requested data, however the statistician also faced similar limitations as was experienced in the initial data retrieval. A future project that is prospective would allow for clinician-driven decision-making in both the data retrieval and analysis, but such a project would also require significant resources to complete compared to the current retrospective design. Alternatively, if LAE becomes a standardized diagnosis with an assigned ICD code, then that code could be used to track more comprehensive data retrospectively. However, a new code would take time to gain acceptance and be utilized widely in documentation. 

Future investigations

Further research may elucidate whether LAE alone or in the setting of comorbidities warrants universal screening practices or prophylactic therapies to prevent cases of PCS. LAE can serve as a therapeutic target for determining when to start treatment with oral anticoagulation, a concept that requires further testing through randomized control trials. Lastly, future studies may also compare the benefits of preventative anticoagulation in patients with LAE with the risk of major bleeding events regarding the utility of acetylsalicylic acid and/or anticoagulants.

## Conclusions

LAE is often found in patients with cardiovascular disease. The results of our study show that LAE is highly prevalent in our population of patients who suffered cryptogenic strokes without atrial fibrillation. LAE also appears to be as prevalent as other known risk factors, such as diabetes. Our findings confirm the significance of LAE. However, further research is needed to compare cardiovascular outcomes in patients with LAE, perhaps utilizing more quantitative methods, such as cardiac CT or MRI, as they become more widely available. Our data also shows that when comparing the various guidelines for LAE measurement on echocardiography, a significant population of patients who suffered strokes did not meet the higher thresholds of LAE. Thus, contemporary research in stroke prevention may be underestimating the prevalence of LAE. Lastly, integrating LAE into patient assessments as an independent diagnosis may play a crucial role in guiding care by improving clinical decision-making methods, such as developing novel scoring systems to assess the value of prophylactic anticoagulation for stroke prevention. 

## Appendices

Appendix A

ICD-10 Diagnosis Codes

A. Hypertension

1. Essential hypertension: I10

2. Hypertensive urgency: I16.0

3. Secondary hypertension: I15.9

4. Hypertensive emergency: I16.1

5. Hypertensive heart disease with chronic kidney disease: I13.0

6. Gestational pregnancy-induced hypertension: O13.9

B. Diabetes mellitus: E11

1. Type II diabetes mellitus without complications: E11.9

2. Type II diabetes mellitus with foot ulcers: E11.621

3. Type II diabetes mellitus with chronic kidney disease: E11.22

4. Long-term current use of insulin: Z79.4

5. Long-term use of oral hypoglycemic drugs: Z79.84

6. Presence of insulin pumps: Z96.41

C. Obesity: E66.0

D. Hyperlipidemia: E78.2, E78.4, E78.5

E. Tobacco use: Z72.0

F. Carotid artery stenosis or atherosclerosis: I65.23

G. Atrial fibrillation: I48

H. Atrial flutter: I48.3

I. Patent foramen ovale: Q21.1

J. Previous transient ischemic attack or cerebrovascular accident: Z86.73

K. Cerebral infarction: I63.9

L. Pulmonary embolism: I26

M. Deep vein thrombosis: I82.40

N. Hypercoagulable states: D68.69/68.59

O. Malignant neoplasm: C80.1

P. Infective Endocarditis: I33.0

Appendix B

CPT Codes

A. Electrocardiogram: 93010

B. Echocardiogram: 93306, 93307

C. Magnetic resonance imaging of the head or brain: 70544, 70551, 70553  

D. Computed tomographic angiography of the head or neck: 70496, 70498

E. Duplex scan of extracranial arteries (carotid ultrasounds): 93880 
